# Blood Lead Concentrations in Newark Children. Comment on Franklin, R.C.; Behmer Hansen, R.A.; Pierce, J.M.; Tsitouras, D.J.; Mazzola, C.A. Broken Promises to the People of Newark: A Historical Review of the Newark Uprising, the Newark Agreements, and Rutgers New Jersey Medical School’s Commitments to Newark. *Int. J. Environ. Res. Public Health* 2021, *18*, 2117.

**DOI:** 10.3390/ijerph18062887

**Published:** 2021-03-12

**Authors:** James M. Oleske, John D. Bogden

**Affiliations:** 1 Department of Pediatrics, Rutgers New Jersey Medical School, Newark, NJ 07102, USA; 2 Department of Microbiology, Biochemistry, and Molecular Genetics, Rutgers New Jersey Medical School, Newark, NJ 07102, USA; john.bogden@rutgers.edu

The recently published article of RC Franklin et al. in the *International Journal of Environmental Research and Public Health* [1] provides a detailed historical review, beginning with the decades of the 1950s and 1960s, of health disparities of Black and Hispanic Newark, New Jersey residents that included substance abuse and sexually transmitted infections, as well as the highest incidence of tuberculosis and maternal and infant mortality in the United States. The manuscript also notes that current Newark residents continue to experience a high incidence of chronic diseases and deficiencies that include food insecurity, hypertension and strokes, diabetes, coronary artery disease, and loss of all their teeth. The article, however, does not describe another serious and glaring health disparity, the long history of high past and current blood–lead concentrations and lead poisoning in Newark children.

One author of this article (JDB) started work as a postdoctoral researcher at New Jersey Medical School in 1971, and was responsible for developing the initial “Lead Laboratory”. This laboratory analyzed blood samples of Newark children 5 days a week, with 50–100 + samples received daily; many were “stat” samples. The other author (JMO) was a recent New Jersey Medical School graduate beginning a residency in Pediatrics at the Martland Medical Center; his inpatients included children hospitalized with lead poisoning. The high blood–lead levels (BLLs) of children revealed by our laboratory analyses at that time were primarily the result of ingestion of small paint chips from peeling indoor paint and/or inhalation of indoor lead-containing paint dust. A key factor was a lack of adequate maintenance and remediation/renovation of painted surfaces in Newark housing rented by low-income families. Inhalation of airborne lead from automobile exhaust was also a major source of exposure for both children and adults. Lead is a cumulative toxin and continued daily exposure can eventually result in elevated BLLs with significant negative multi-organ system health consequences, including hematological and neurological abnormalities. A significant percent of Newark children during this era had elevated blood–lead concentrations high enough to require emergency hospitalization for multi-organ system abnormalities that even became life-threatening in scope. Lead is a neurotoxin and many of these children were diagnosed with mild cognitive dysfunction and even more severe permanent brain damage.

Although other large USA cities in the Northeast (Philadelphia, Baltimore, New York City) also had poorly maintained housing and many children with lead poisoning, Newark had an especially severe problem [2], with 41.2% of 25,260 mostly Newark children tested between 1970 and 1976 having BLLs ≥30 mcg/dL. Of these 15.6% had high BLLs ≥ 40 mcg/dL and 1.8% had dangerously high BLLs ≥60 mcg/dL that typically required hospitalization for observation for toxicity and intravenous pharmacologic therapy. The substantial number of Newark children found to have high BLLs during the early 1970s may be explained by the older age of much Newark housing, inadequate maintenance efforts to prevent and remove peeling and flaking paint, and our extensive testing of children’s BLLs—enabled by substantial funding. In contrast, more than 95% of young New Jersey children currently have BLLs less than 2.0 mcg/dL, but we could not find a single Newark child in the 1970s with a BLL below 5.0 mcg/dL.

The lead crisis of the 1960s and 1970s would have been worse without the dedicated care provided by Newark pediatricians at St Michael’s Medical Center, Newark Beth Israel Medical Center, and our faculty at Martland Hospital. A clinical challenge in the 1970s for pediatricians was the collection of an adequate volume (1.5 mL or more) of venous blood for lead analysis from screaming children in the presence of their anxious parents —most often the mothers, but sometimes the more threatening fathers. Handing out lollipops helped, but it was the skill and rapidity of the blood drawer as well as the good work of the holder of the squirming/moving/crying child that saved the day.

New Jersey law requires pediatricians to order testing of BLLs of all children at both 12 and 24 months of age, and also prior to age 6 for all children not tested when younger. Children with known or suspected lead exposure should also be tested. Compliance with these regulations in New Jersey has been very good, with 86% of these children tested in 2018. This testing reveals that, although the mean BLLs of Newark and other New Jersey children are much lower now [3], there are still New Jersey children with elevated BLLs greater than the current guideline of 5.0 mcg/dL.

As an element, lead cannot decompose, and thus has an infinite environmental half-life. It was present in paint used in almost all older homes built in the United States before the 1970s. Although much of this housing in Newark and elsewhere in the United States has been professionally “de-leaded”, in other housing the lead is still there in lower layers of dried paint on painted surfaces such as walls and windows, where it is especially prone to flaking and chipping. The city of Newark is using funding from a US Department of Housing and Urban Development (HUD) grant to continue abatement/removal of lead paint in housing and is also finishing its effort to the replace about 18,000 “lead service” plumbing lines to provide lead-free water to many of Newark’s houses.

In older housing that has not undergone lead abatement, layers of dried lead paint will still be on painted surfaces dozens and even hundreds of years from now. Thus, lead exposure will continue to be a threat to young children and the need to test them for elevated BLLs should be recognized and continued.
